# An extremely rare case of indirect hernia type co-existing with testicular ectopia

**DOI:** 10.11604/pamj.2020.35.119.21129

**Published:** 2020-04-14

**Authors:** Christos Plataras, Ioannis Alexandrou, George Bourikis, Dimitris Bourikas, Efstratios Christianakis

**Affiliations:** 1 Pediatric Surgery Clinic, Penteli's Children Hospital, Attiki, Greece; 2 General Surgery Department, Tzanio Hospital, Piraeus, Greece

**Keywords:** Hernia, child, undescended testis

## Abstract

We present an extremely rare case of inguinal hernia coexisting with testicular ectopia in a child. Male infant 9.5 month old presented with an empty scrotum and the ipsilateral intravaginal testis lying in a high iliac crest position. When crying a moving right inguinal bulge appeared on clinical examination. This grew bigger in moments of increased abdominal pressure and seemed to move upwards towards the right ileac crest. No abdominal wall defect could be palpated. At operation a large hernia sac fixed in the area of the right iliac crest was identified. Adjacent was the fixation point of the gubernaculum and the testis was found in an ectopic location. We removed the large sac after separating the vas and vessels and the testis and we strengthened the dorsal inguinal wall and fixed the testis in a subdartos scrotal pouch. No postoperative complications happened. An undescended testis may present as an iliac crest ectopy, coexisting with moving inguinal hernia. In our case we propose that the higher position of the aponeurosis of the external oblique in combination with ectopia of gubernacular fixation in the ipsilateral scrotum may have caused the ectopic fixation of the sac in the ipsilateral inguinal crest.

## Introduction

The cause of indirect hernia is the presence of a protruding peritoneal sac (patent processus vaginalis) at the deep inguinal ring. The deep inguinal ring is formed basically by aponeurotic bars of the transversalis fascia sling, with the inferior border of the ring formed by the iliopubic tract, its superior border formed by the transversus abdominis arch and its median border formed by the inferior epigastric vein [1]. Undescended testis occurs in 3% of term infant boys and in up to 33% of premature boys. The majority of testes descend within the first 9 to 12 months. At age 1 year, the incidence of undescended testis is 1% 21. Anomalies associated with undescended testis include patent processus vaginalis, inguinal hernia, hypospadias, posterior urethral valve and anomalies of the upper urinary tract [2, 3]. Anatomic variations may predispose to both inguinal hernia and undescended testis. We present a rare variant of a combination of inguinal hernia and ectopic testis that presented as a mobile inguinal mass in the right lower abdominal area.

## Patient and observation

**Figure 1:**
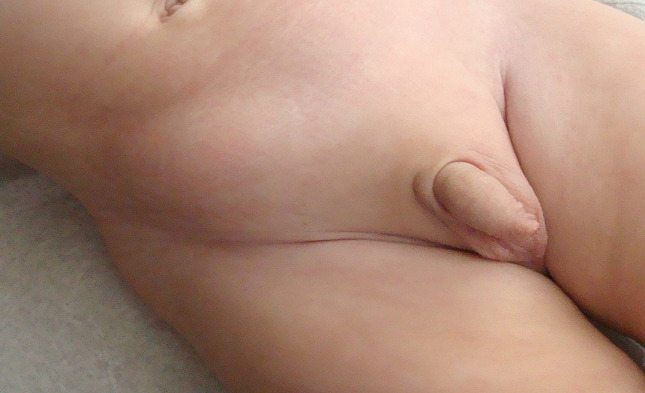
Right inguinal bulging: the mass increased in size in moments of increased abdominal pressure especially when crying and seemed to move upwards towards the right abdominal area

**Figure 2:**
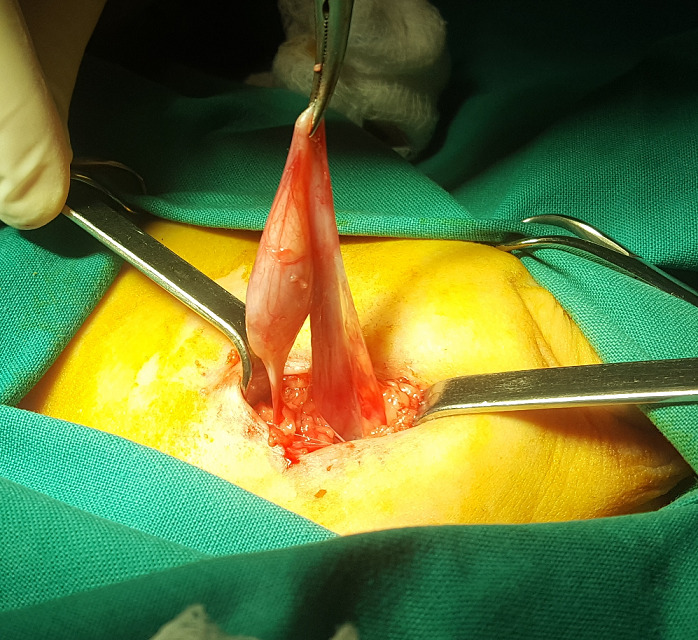
The hernia sac and the testis can be seen: the sac was fixated in the area of the right inguinal crest and the vas and the vessels of the ipsilateral testis were intravaginal

Male infant 9.5 month old was examined in the emergency department of our hospital because of an empty scrotum and the ipsilateral testis lying in a high iliac crest position. With the increase in intrabdominal pressure [crying etc.] there was an ever-increasing swelling with spiral course from the groin to ipsilateral iliac crest. On clinical examination, a reducible mass was present in the right ileac crest (Figure 1). The swelling looked like a spigelian hernia [4]. However no abdominal wall defect could be palpated at that area. Moreover the ipsilateral scrotum was empty and the testis could be palpated with difficulty lying in a high inguinal position. Ultrasonography showed a loop of herniated small bowel and an undescended testis on the right. At operation, under general anesthesia, an extended transverse inguinal incision was made. Upon entering the inguinal canal a large hernia sac was identified. This followed a course through the superficial inguinal ring, extended upwards to the anterior abdomninal wall below the subcutaneous plane and was fixed in the area of the right iliac crest. We noticed that the hernia orifice was in a higher than normal position 5 cm from the pubic bone. This bulk was easily reduced back to the abdomen along with an ipsilateral mobile intravaginal testis. After careful isolation of the sac at a length of 3 cm the ipsilateral testis was isolated. The vas and the vessels were intravaginal, the gubernaculum was fixed in an ectopic position (Figure 2) and the dorsal inguinal wall was lax. We removed the large sac after separating the vas and vessels and the testis. Moreover we strengthened the dorsal inguinal wall, fixated the testis in a subdartos pouch and closed the wound. Patient stayed for a day in the hospital. No postoperative complications happened. Three years later the boy was operated for left hydrocele. The boy remains in perfect health now five years after the initial operation.

## Discussion

An undescended testis may present as an iliac crest ectopy. This is an uncommon presentation compared to undescended testis. There are also reports of “intraparietal hernia” and “abdominal wall ectopic testis”. Ectopic testes are thought to be greater at risk of trauma, testicular torsion, sub-fertility and malignancy [5-7]. Our patient seems to belong to this group of hernias. In our case an inguinal mass was palpated and seemed to move laterally with an upward direction away from the scrotum which is not the usual finding in a case of undescended testis. At least 80-90% of testes are palpable in the inguinal region or can be squeezed out of the inguinal canal and felt at the external ring by pressing firmly on the abdominal wall laterally near the anterosuperior iliac spine and pressing downward and medially toward the scrotum [8]. This manoeuvre was not possible in our case. Testicular descent occurs in two phases: a transabdominal phase and an inguinoscrotal phase. The 1^st^ phase, the transabdominal phase (8-15 weeks of gestation) is controlled by enlargement of the gubernaculum and regression of the cranial ligament. Insulin-like hormone 3 is the primary regulator of the 1^st^ phase, possibly assisted by mullerian inhibiting substance/antimullerian hormone (MIS/AMH) and by regression of the cranial suspensory ligament induced by testosterone. The second phase (25-35 weeks of gestation), the inguinoscrotal phase, requires migration of the gubernaculums from the groin into the scrotum and its migration is guided by calcitonin gene-related peptide released by the genitofemoral nerve. The inguinoscrotal phase of testicular descent is regulated by androgens and by calcitonin gene-related peptide release by the sensory nucleus of the genitofemoral nerve [9-11]. The pathogenetic mechanism of typical indirect inguinal hernias involves expansion of the hernia sac [patent processus vaginalis] in the inguinoscrotal direction through the inguinal canal by abdominal pressure. As a result the hernia sac has direction towards or may even reach the scrotum [6]. In our case there was ectopic fixation of the gubernaculum and we propose that a defect in the first phase of testicular descent prevented normal development of the gubernaculum. That combined with the higher position of the aponeurosis of the external oblique in combination with gubernaculum ectopia in the ipsilateral scrotum may have caused the ectopic fixation of the sac in the ipsilateral inguinal crest. In our case the bulging hernia sac had a direction toward the right lower abdominal wall.

## Conclusion

In conclusion perhaps the most common operative procedures in infants and young children involve the inguinal area for the repair of hernia, hydrocele and undescended testicle. Many of the unexpected findings in such operations are peculiar to children [12]. An awareness of the possibility of such surprises is essential to physicians who do inguinal operations in pediatric age groups.

## Competing interests

The authors declare no competing interests.
